# Efficacy of a Traditional Herbal Formula, *Banha-Sasim-Tang* in Functional Dyspepsia Classified as Excess Pattern

**DOI:** 10.3389/fphar.2021.698887

**Published:** 2021-08-27

**Authors:** Yun Hee Kim, Jun Young Kim, O-Jin Kwon, So Young Jung, Jin-Yong Joung, Chang Sop Yang, Jun-Hwan Lee, Jung-Hyo Cho, Chang-Gue Son

**Affiliations:** ^1^ Korean Medicine Convergence Research Division, Korea Institute of Oriental Medicine (KIOM), Daejeon, Korea; ^2^ Liver and Immunology Research Center, Daejeon Oriental Hospital of Daejeon University, Daejeon, Korea; ^3^ Korean Medicine Clinical Research Division, Korea Institute of Oriental Medicine (KIOM), Daejeon, Korea

**Keywords:** functional dyspepsia, banha-sasim-tang, investigator-initiated, randomized trial, excess pattern

## Abstract

This study evaluated the efficacy and safety of *Banha-sasim-tang* (BST) in patients with functional dyspepsia (FD). BST (*Banxia-xiexin-tang* in traditional Chinese medicine and *Hange-shashin-to* in Kampo medicine) is traditionally prescribed for the treatment of dyspepsia with epigastric stiffness and gastric fullness in China, Japan, and Korea. Patients with FD were randomly administered an oral dose (10 g) of BST syrup or placebo, twice a day for 4 weeks. The primary outcome was the symptom checklist part of the Nepean dyspepsia index (NDI). The secondary outcomes were the quality of life (QoL) part of the NDI, functional dyspepsia-related QoL (FD-QoL), and visual analog scale (VAS). A total of 60 patients with FD were screened, and 50 were randomized into BST group (n = 25) and placebo group (n = 25). Two patients in the placebo group withdrew before the start of the treatment. Administration of BST syrup resulted in improvement in the symptom-related NDI score in the BST group compared with that in the control group; however, the difference was not significant. BST syrup significantly improved “fullness after eating” index of NDI at follow-up time point (2.88 ± 2.65 vs 4.78 ± 2.69, *p* = 0.0081). In the total score of the QoL section of the NDI and FD-QoL scales, there was no significant improvement in the BST group compared to that in the placebo group. With regard to improvement in overall FD symptoms, the VAS scale showed improvement in both groups, but the difference was not significant. Interestingly, follow-up investigation showed a significantly beneficial effect of BST on FD symptoms, when compared to placebo. Significant improvement observed in VAS score (39.60 ± 22.29 vs 52.17 ± 20.55, *p* = 0.048). This indicated that the effect of BST lasted even after the completion of the medication regimen. Overall, our data suggest that while BST showed no significant improvement in the symptom-related NDI score and the QoL related scores in NDI and FD-QoL after 4 weeks of treatment, it effectively improved the VAS score and fullness after eating-related symptoms in the follow-up visit.

**Clinical Trial Registration:**https://cris.nih.go.kr; Identifier KCT 0002013

## Introduction

Functional dyspepsia (FD) refers to the presence of gastrointestinal symptoms, such as postprandial fullness, early satiety, epigastric discomfort, bloating, and nausea (Lacy et al., 2013). Although these symptoms are non-life-threatening and FD is not related to an increase in mortality, relapsing-remitting symptoms of FD cause impairment in the quality of life (QoL), and the symptoms cause decreased productivity and activity in the workplace (Mahadeva and Goh, 2006; Talley and Ford, 2015). Therefore, a quarter of the patients with FD choose to consult a physician, thus adding to the excessive health care costs, which exceed several billion dollars annually in the U.S.(Tack et al., 2004; Talley et al., 2006b; Talley et al., 2012). However, the conventional treatment remains unsatisfactory for many patients, and up to 50% of the patients with FD seek alternative herbal treatments such as STW5, which consists of nine herbs (Lacy et al., 2012; Talley and Ford, 2015).

*Banha-sasim-tang* (BST, *Banxia-xiexin-tang* in traditional Chinese medicine [TCM] and *Hange-shashin-to* in Kampo medicine) is composed of seven herbs as follows: *Pinellia ternate* (Thunb.) Makino., tuber; *Scutellaria baicalensis* Georgi., root; *Panax ginseng* C.A. Mey., root and rhizome; *Glycyrrhiza uralensis* Fisch., root and rhizome; *Ziziphus jujuba* Mill., fruit; *Zingiber officinale* Roscoe., rhizome; *Coptis chinensis* Franch., rhizome (Naito et al., 2003; Organization, 2007; Zhao et al., 2013). BST is traditionally prescribed in China, Japan, and Korea, as the treatment for dyspepsia with epigastric stiffness and gastric fullness. BST is also used for various gastrointestinal (GI) tract disorders such as chronic gastritis and reflux esophagitis (Xia, 2004; Xu, 2006), diarrhea in patients receiving chemotherapy (Gochi et al., 1995; Mori et al., 1998; Kono et al., 2010). The efficacy of BST in gastrointestinal diseases such as functional dyspepsia (Park et al., 2013; Zhao et al., 2013), chronic atrophic gastritis (Cao et al., 2020) and gastroesophageal reflux disease (Dai et al., 2017) has been evaluated in several studies. However, its efficacy of BST is controversial in FD trials. We speculated that these controversial results were due to drug administration in patients without pattern identification considerations based on individual differences and state. The TCM pattern identification is a system of diagnosis used in TCM. According to TCM pattern diagnosis, individuals may show different symptoms and signs depending on their physical conditions and health status even though they may suffer from the same disease. Different symptoms and signs are classified into several “patterns” (called “Zeung” in Chinese), which have been used as diagnostic tools and for prescribing medicinal herbs and acupoints in TCM (Jiang, 2005; Berle et al., 2010; van der Greef et al., 2010; Zhang et al., 2012).

With regard to FD, herbal formulas such as BST, *Yukgunja-Tang*, and *Hyangsa-Pyeongwi san* (Kim et al., 2014; Kim et al., 2017) are prescribed by Korean medicine doctors depending on the differentiation of the patient’s symptoms and signs according to diagnostic patterns in Korea. BST was recorded in the old Chinese medical literature, *Shan-han-za-bing-lin* (傷寒雜病論). The indication of BST in *Shan-han-za-bing-lin* is “Epigastric stiffness (心下痞)” which is similar to the epigastric or upper abdominal fullness (World Health Organization, 2007; Jeon et al., 2019). In this randomized, blinded, parallel-group trial, we classified FD patients with epigastric stiffness and other related signs as “excess pattern” using a questionnaire (Kim et al., 2010) and specifically evaluated the efficacy and safety of BST syrup for the treatment of FD symptoms.

## Materials and Methods

### Trial Oversight

This trial was an investigator-initiated, randomized, blinded, parallel-group trial of BST syrup, manufactured by Jeong-woo Pharmaceutical Company Ltd., (Seoul, Korea) versus placebo for patients with FD in the Daejeon Korean Medicine Hospital in Daejeon, Korea. The study was performed according to the rules, guidelines, and regulations of the Ministry of Food and Drug Safety of the Republic of Korea and was approved by the Institutional Review Board of Daejeon Oriental Medical Center (Authorization no.: djomc-136-01). It was registered in the Clinical Research Information Service of the Republic of Korea (KCT 0002013). From July 15th, 2016 to December 31st, 2016, a total of 50 patients were informed about the trial and provided written consent for participation. All subjects provided written informed consent in accordance with the Declaration of Helsinki.

### Participants

Patients aged between 19 and 75 years with dyspepsia symptoms were eligible for enrolment in the study. Eligible subjects met the ROME III criteria (Drossman and Dumitrascu, 2006) and had one or more of the following symptoms: early satiety, postprandial fullness, and epigastric pain or burning that was not due to structural disease in medical assessment (including upper endoscopy) for more than 3 months and an onset of symptoms at least 6 months prior to study enrollment. Additionally, the included patients had two or more of the following eight moderate symptoms: epigastric pain, discomfort, stuffiness, fullness, burning, postprandial fullness, early satiety, and nausea and a total of at least 6 points in the scoring of the eight symptoms using the following severity scale: 0, mild; 1, moderate; 2 and 3, severe. Subsequently, eligible subjects met an excess pattern, as determined using an instrument of pattern identification for FD (Kim et al., 2010). The patient exclusion criteria were a history of gastrointestinal surgery (except appendectomy), the presence of gastrointestinal bleeding, intestinal obstruction, gastrointestinal perforation, colorectal cancer, duodenitis, or stomach cancer. Moreover, patients with severe hepatic dysfunction, congestive heart failure, renal failure, and elevation over the normal range of liver enzymes observed two or more times were excluded. Additional exclusion criteria were current or past use of non-steroidal anti-inflammatory drugs, corticosteroids, or other investigational study drugs within 30 days before study entry, or pregnancy or lactation in women. All participants fully comprehended the purpose of this trial and the risks involved before participation and submitted their informed consent to participate in the study. Finally, a total of 50 patients with FD were enrolled (25 in the BST group and 25 in the placebo group).

### Study Design

**FIGURE 1:**
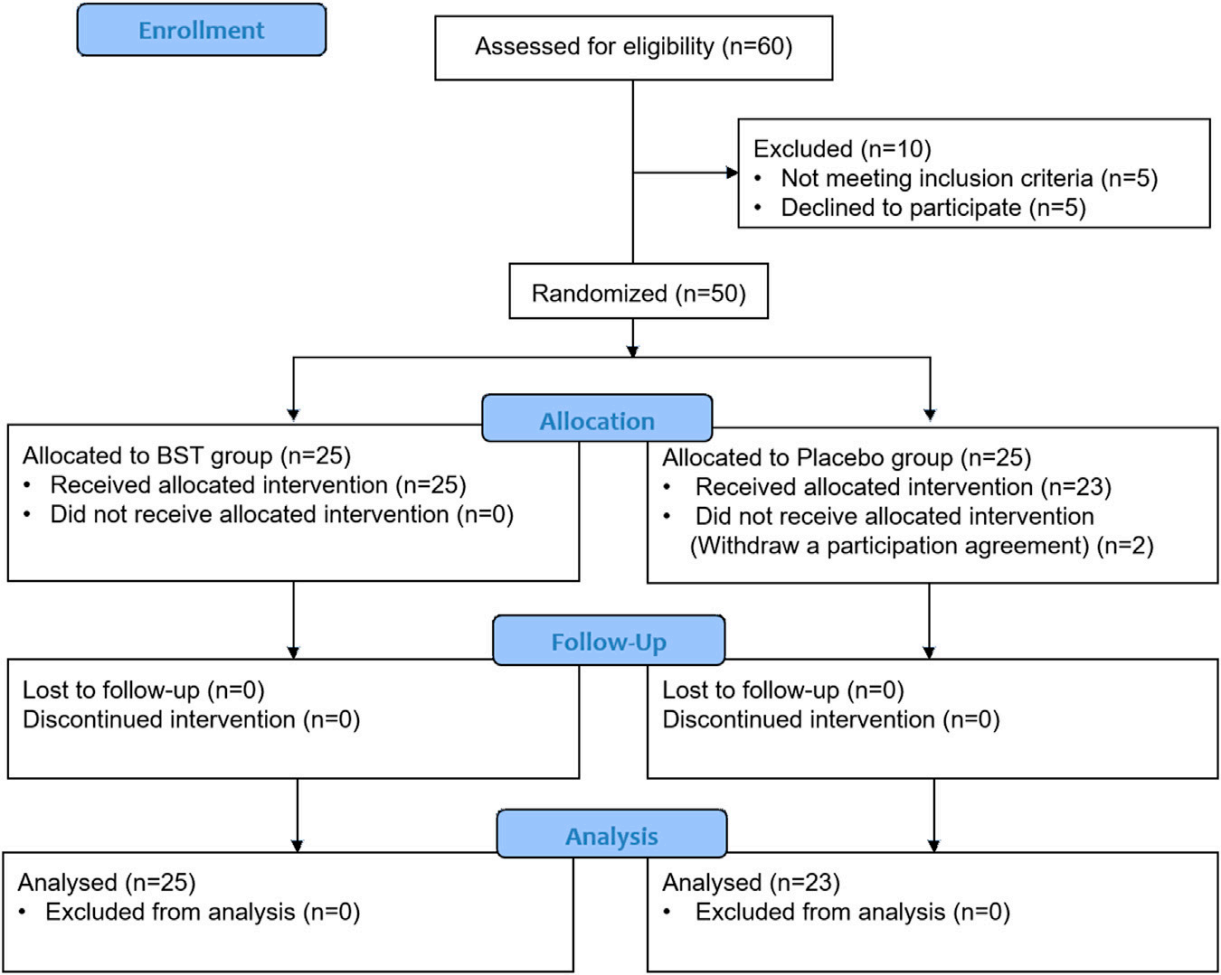
Flow diagram of subject progress through the phase of the RCT.

This study is an investigator-initiated, randomized, blinded, parallel-group trial of BST syrup versus placebo in patients with FD. This clinical trial consisted of 4 weeks of oral administration of BST syrup or placebo, and a 4-weeks follow-up period. Thus, the participants made four visits after the screening. Participants were randomly assigned to two groups: BST syrup or placebo. BST syrup or placebo (20 g each) was administered orally to patients in each group (a single dose was 10 g). The random sequence was generated using a computer-generated random number table, and blocked randomization was applied. The investigators, participants, and research sites were blinded to the study group assignment and treatment. Participants were administered BST syrup or placebo twice daily for 4 weeks. After 2 weeks of ingesting BST syrup or placebo, participants visited the clinical center where the leftover BST syrup or placebo was checked to measure compliance. The participants were then given BST syrup or placebo for the remaining 2 weeks. The participants and clinicians were blinded until the end of the study. To evaluate the success of blinding, at the end of the trial, patients were asked about their opinion whether they regarded the drug administered as the active drug or placebo. The whole process of clinical trial is summarized in Figure 1.

### Preparation of Drugs and Fingerprinting Analysis

**FIGURE 2:**
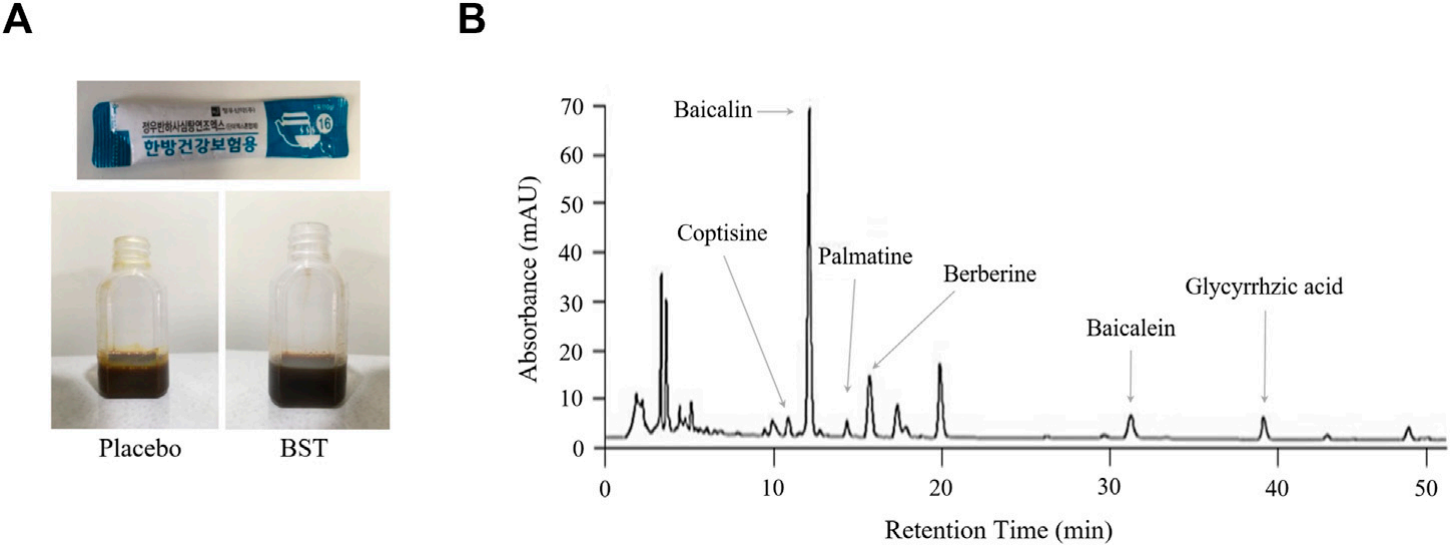
Fingerprint analysis. The real images of placebo and BST syrup **(A)** and *Banha-sasim-tang* (BST) using ultra-high-performance liquid chromatography-tandem mass spectrometry (UHPLC-MS/MS) **(B)**.

BST syrup (17JW-08), prepared by Jeong-woo Pharmaceutical Company Ltd. (Seoul, Korea), was produced according to the Korean Good Manufacturing Practice. The BST soft extract was composed of the following herbs: Pinelliae tuber (*Pinellia ternate* (Thunb.) Makino, 1.178 g), Scutellariae radix (*Scutellaria baicalensis* Georgi, 0.840 g), Ginseng radix (*Panax ginseng* C. A. Mey., 0.803 g), Glycyrrhizae radix (*Glycyrrhiza uralensis* Fisch., 0.732 g), Zizyphi fructus (*Ziziphus jujuba* Mill., 0.512 g), Zingiberis rhizoma (*Zingiber officinale* Roscoe, 0.500 g), Coptidis rhizoma (*Coptis chinensis* Franch., 0.133 g), and Zingiberis rhizoma recens (*Zingiber officinale* Roscoe, 0.077 g). All herbal materials were extracted in boiling water, and the extracts were filtered, concentrated, and lyophilized. Following the lyophilized, four additives: β-Cyclodextrin (1.500 g), apple concentrate (1.350 g), carboxymethyl cellulose (50 mg), and sodium benzoate (5.4 mg) with water were added. The matching placebo was provided by the same supplier and was similar in form, flavor, and fragrance to BST syrup, and contained starch (0.600 g), lactose mixture (1.412 g), and caramel food coloring (Figure 2A).

To validate the quality of BST syrup, fingerprinting analyses of BST (Figure 2B) and six reference compounds of each herbs (coptisine, baicalein, glycyrrhizic acid, palmatine, berberine, and baicalin) were conducted using high-performance liquid chromatography (HPLC, Agilent 1,100 system, CA, United States), equipped with an auto sampler (G11313A), column oven (GA1316A), binary pump (G1312), diode-array-detector (DAD), and degasser (GA1379A), as described previously (Jeon et al., 2019).

### Assessment of Efficacy

The effect of BST syrup was evaluated using a symptom-based questionnaire with the Nepean dyspepsia index (NDI) (Talley et al., 1999). The NDI consists of two distinct instruments: a symptom checklist and a disease-specific QoL measure. The symptom checklist part of the NDI was used as the primary outcome evaluation. Frequency (scored 0–4), intensity (scored 0–5), and bothersomeness (scored 0–4) were rated according to the state of the symptoms. Higher NDI symptom scores indicated the severity of FD symptoms. Secondary outcome measures consisted of the following items: the total score of QoL section of the NDI, which consists of 25 items; the total score of the validated 21 items of FD-related QoL (FD-QoL) scale [21]; and a change from the baseline in the total score of the visual analog scale (VAS) for measuring severity or pain. We rated each item of the NDI QoL without weighting. We reversely rated the QoL scores so that higher QoL scores account for higher QoL. All outcomes were assessed at days 0, 2, and 4 during BST administration and at 4 weeks after completion of BST administration.

### Safety Assessment

All participants were required to report any adverse events during the trial. Hematological tests for liver and renal functions, vital sign measurements, and electrocardiography were conducted on the second and fourth visits.

### Sample Size Calculation

The purpose of this study was to evaluate the efficacy of BST treatment on FD. In this trial, the sample size was estimated based on the clinical experiences and the results of two previous studies (Lee and Kim, 2013; Kim et al., 2014). The average variation (*δ*) was assumed to be 20 and the standard deviation (SD = σ) was assumed to be 22.4 between the two groups. The patients were randomized in a 1:1 ratio to each arm. Results were assessed using a 2-sided test with a statistical power of 80% and a significance level (α) at 5%. Therefore, this trial included 40 participants, who were divided into two groups of 20 (*n*) each, as determined using the following equation:

$$\text{n}=\frac{2\times {\left(z_{\alpha /2\;}+z_{\beta }\right)}^{2}\times \sigma^{2}}{\delta^{2}}$$

Assuming a potential dropout rate of 20%, 25 participants were required to be recruited in each group, totaling 50 participants.

### Statistical Analysis

Statistical analysis was performed using the statistical analysis SAS software (version 9.4, SAS Institute Inc., Cary, NC, United States), and each individual was considered as a unit of analysis. Data were analyzed using intention-to-treat analysis, and all participants who received treatments after randomization were included. All reported *p*-values are two-tailed, and for each analysis, statistical significance was set at *p* < 0.05, and data are described as the mean ± standard deviation (SD). Categorical variables such as baseline clinical characteristics were compared using Fisher’s exact test. Continuous variables were compared using ANCOVA test and independent *t*-test for normally distributed data and Wilcoxon rank-sum test for data with non-normal distribution.

## Results

### Patient Disposition and Baseline Characteristics

The trial was performed from July 2016 to December 2016, and a total of 60 candidates were screened. A total of 50 subjects were matched to the inclusion criteria and randomized into two groups, the BST group and the placebo group, at a one-to-one ratio. Two participants in the placebo group dropped out owing to personal reasons before starting the first treatment and were excluded from the modified intention-to-treat (ITT) analysis. Therefore, 23 participants in the placebo group and 25 in the BST group were included in the modified ITT analysis. The baseline characteristics were similar between the two groups, as shown in Table 1.

**TABLE 1:** Participants baseline characteristics.

	Treatment group (n = 25)	Control group (n = 23)	*p*-value
Gender (Male/Female)^a^	8 (32.0%)/17 (68.0%)	5 (21.7%)/18 (78.3%)	0.5234
Age (year)^b^	50.96 ± 10.63	49.22 ± 11.43	0.5947
Height (cm)^b^	162.12 ± 6.82	159.55 ± 7.70	0.2357
Body weight (kg)^b^	62.54 ± 8.40	60.18 ± 13.21	0.4798
BMI (kg/m2)^b^	23.78 ± 2.62	23.45 ± 3.78	0.7187
Smoking (Yes/No)^a^	1 (4.0%)/24 (96.0%)	1 (4.4%)/22 (95.6%)	0.9999
Drinking (Yes/No)^a^	10 (40.0%)/15 (60.0%)	4 (17.4%)/19 (82.6%)	0.1167
Caffeine (Yes/No)^a^	17 (68.0%)/8 (32.0%)	16 (69.6%)/7 (30.4%)	0.9999

a Fisher’s exact test.

b Student’s independent *t*-test.

### Efficacy

BST administration improved the NDI score in the BST group compared to that in the placebo group after 4 weeks; however, the difference was not significant (*p* = 0.3102, Table 2). Nevertheless, BST did not improve the NDI QoL, FD-QoL (*p* = 0.4358, *p* = 0.0926, Table 3), and VAS scores (*p* = 0.1052, Table 4).

**TABLE 2:** Symptom scores using Nepean Dyspepsia Index (NDI).

Items and domains	Placebo		*Banha-sasim-tang (BST)*		*p*-Value^a^
	0 weeks	4 weeks	0 weeks	4 weeks	
**Symptoms**
Pain in upper abdomen	4.69 ± 3.16	3.22 ± 2.56	5.32 ± 3.36	2.68 ± 2.90	0.4234
Discomfort in upper abdomen	5.26 ± 3.0	2.83 ± 2.48	4.92 ± 32.7	2.84 ± 2.97	0.8329
Nausea	3.60 ± 2.91	2.09 ± 3.10	3.56 ± 3.42	1.04 ± 1.65	0.1282
Inability to finish a regular meal	4.95 ± 3.33	3.09 ± 3.16	3.44 ± 2.73	1.96 ± 1.46	0.1914
Fullness after eating	6.43 ± 2.72	4.52 ± 3.19	6.8 ± 2.95	3.16 ± 1.40	0.0247^a^
Pressure in upper abdomen	6.47 ± 2.53	3.78 ± 2.37	5.56 ± 3.06	3.08 ± 3.11	0.6543
Burning in upper abdomen	4.47 ± 3.69	4.04 ± 3.30	4.2 ± 2.73	2.48 ± 2.83	0.0823
Heart burn	3.82 ± 3.21	2.87 ± 3.09	3 ± 2.78	2.52 ± 3.07	0.949
Bitter tasting fluid taste	4.95 ± 3.67	3.09 ± 3.15	3.32 ± 3.35	2.24 ± 2.80	0.723
Burping	6.95 ± 3.75	4.96 ± 3.46	5.76 ± 3.45	3.48 ± 3.15	0.3082
Cramps in upper abdomen	2.39 ± 3.11	1.65 ± 2.71	1.8 ± 3.36	1.32 ± 3.04	0.8914
Chest pain	3.21 ± 2.98	2.65 ± 3.02	3.48 ± 3.41	1.96 ± 2.96	0.3186
Vomiting	1.17 ± 2.12	1.13 ± 2.03	1.88 ± 3.28	0.76 ± 1.90	0.4945
Bad breath	2.47 ± 2.79	1.30 ± 1.96	1.68 ± 2.86	0.92 ± 1.53	0.6667
Bloating in upper abdomen	6.65 ± 2.88	3.52 ± 2.84	6.52 ± 3.25	3.28 ± 1.93	0.7544
Total score	67.57 ± 26.75	44.74 ± 31.76	61.24 ± 25.89	33.72 ± 26.40	0.3102

a *p*-value by Analysis of covariance (ANCOVA).

**TABLE 3:** Quality of Life (QoL) scores using NDI and FD-QoL.

Items and domains	Placebo		*Babha-sasim-tang (BST)*		*p*-value^a^
	0 weeks	4 weeks	0 weeks	4 weeks	
NDI-QOL
Interference	51.73 ± 12.49	64.09 ± 14.20	52.47 ± 12.26	61.97 ± 18.01	0.5752
Knowledge/Control	49.49 ± 11.43	64.57 ± 14.63	52.62 ± 13.36	63.29 ± 19.89	0.4582
Eat/drink	57.28 ± 20.16	68.46 ± 17.43	60.21 ± 16.60	67.41 ± 18.71	0.6057
Sleep disturbance	59.65 ± 14.71	73.91 ± 13.20	65.68 ± 20.29	70.40 ± 20.72	0.4063
Total score	54.54 ± 12.12	67.76 ± 11.84	57.75 ± 11.26	65.77 ± 17.08	0.4358
FD-QOL					
Eating	58.48 ± 18.80	72.83 ± 17.63	69.00 ± 16.07	81.00 ± 12.83	0.2063
Liveliness	50.27 ± 17.92	67.93 ± 19.52	59.00 ± 18.58	77.00 ± 13.59	0.1094
Psychological	63.95 ± 22.46	80.07 ± 20.41	75.67 ± 16.95	87.00 ± 10.85	0.3447
Role-functioning	67.93 ± 18.35	84.06 ± 14.14	78.83 ± 15.77	89.00 ± 10.48	0.3512
Total score	60.16 ± 15.79	76.22 ± 15.05	70.62 ± 12.52	83.50 ± 9.74	0.0926

a *p*-value by Analysis of covariance (ANCOVA).

**TABLE 4:** Visual Analogue Scale (VAS) scores.

Items and domains	Placebo		BST		*p*-value^a^
	0 weeks	4 weeks	0 weeks	4 weeks	
**VAS**
Interference	62.04 ± 17.99	45.13 ± 23.22	64.32 ± 15.99	34.92 ± 17.83	0.1052

a *p*-value by Analysis of covariance (ANCOVA).

### Follow-Up Study

**FIGURE 3:**
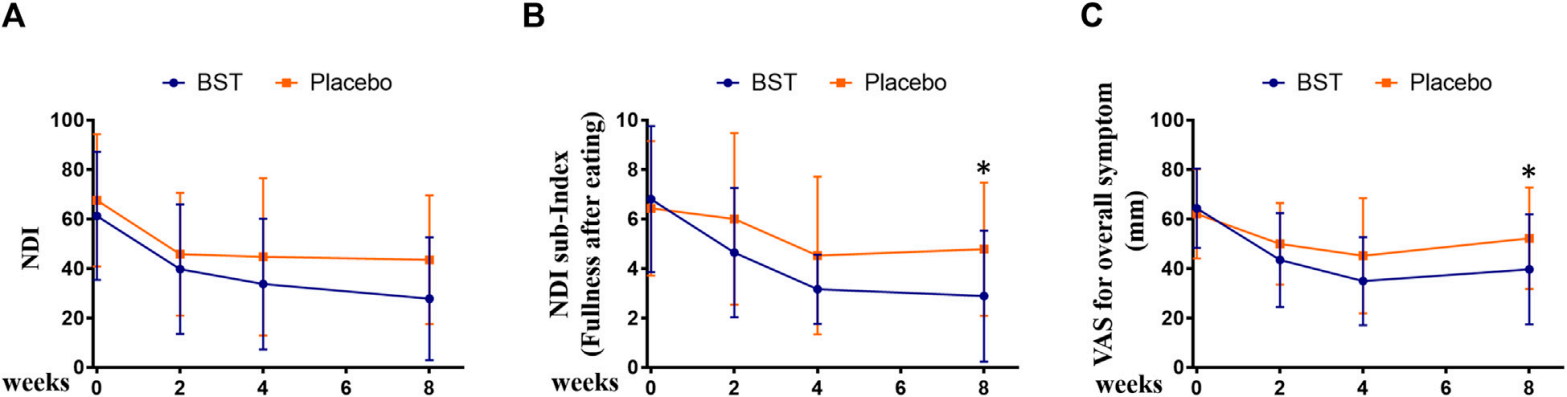
Changes in outcomes between *Banha-sasim-tang* (BST) and placebo group during the trial. **(A)** Changes in the Nepean Dyspepsia Index (NDI) symptom score. **(B)** Changes in the sub-index of NDI symptom score. **(C)** Changes in the visual analog scale (VAS) for overall symptoms. *p < 0.05 vs placebo group.

To evaluate the long-lasting effect of BST after administration, outcomes were measured at the follow-up visit, that is, 4 weeks after the final treatment. BST treatment improved the NDI score at follow-up when compared with that observed for the placebo group, with a marginal trend toward significance (27.76 ± 24.86 vs 43.52 ± 26.01, *p* = 0.0569, Figure 3A). The score of “fullness after eating” in the NDI index decreased significantly after BST treatment as compared to the placebo group at follow-up (2.88 ± 2.65 vs 4.78 ± 2.69, Wilcoxon rank-sum test, *p* = 0.0081, Figure 3B). Furthermore, oral administration of BST caused a significant change in VAS compared to that in the placebo group (39.60 ± 22.29 vs 52.17 ± 20.55, *p* = 0.048, Figure 3C).

### Safety

No serious adverse events occurred during the trial. Eight cases of mild adverse events were reported in the BST group, which were as follows: nausea (3 cases), headache (1 case), abdominal pain (1 case), epigastric fullness (1 case), urticaria (1 case), and acid reflux (1 case). Nine cases of mild adverse events, including nausea (2 cases), vomiting (1 case), headache (1 case), abdominal pain (1 case), diarrhea (1 case), dyspepsia (1 case), and slight elevation in aspartate transaminase or alanine aminotransferase levels (2 cases) were reported in the placebo group.

## Discussion

Dyspepsia, defined as pain or discomfort referable to the gastroduodenal region of the gastrointestinal tract, occurs very commonly in the general population. Epidemiological studies have shown that 15–20% of the population in Western countries has dyspepsia in the course of 1 year (Tack et al., 2004). In studies using upper gastrointestinal endoscopy, more than 70% of the patients with dyspepsia had FD, while less than 10% and less than 1% of the patients with dyspepsia had a peptic ulcer and gastroesophageal cancer, respectively (Ford et al., 2010). Despite the high prevalence of FD, the golden strategy for the treatment of FD remains incomplete, and thus many patients with FD choose other types of treatment. A survey study showed that 36.7% of the patients with functional gastrointestinal disorders used herbal drugs herbal drugs as an alternative or complementary medicine (Lahner et al., 2013; Ottillinger et al., 2013). BST is a TCM that has been widely used for treating gastrointestinal disease. Both clinical and preclinical studies have shown the beneficial effects of BST for FD (Gan et al., 2014; Jeon et al., 2019).

In this investigator-initiated, randomized, blinded, parallel-group trial, we aimed to evaluate the efficacy of the herbal drug BST syrup. Results from the primary outcome measurement showed that the mean value of NDI total score was 61.24 in the BST group and 67.57 in the placebo group, and after 4 weeks of treatment, the scores of FD symptoms were ameliorated in both the groups (33.72 ± 26.40 versus 44.74 ± 31.76, respectively). In addition, the score of “fullness after eating” in the NDI index significantly decreased after BST treatment at follow-up. This improvement was consistent with the findings of previous studies that showed that BST improved GI motility and increased gut motility hormone levels (Park et al., 2013; Jeon et al., 2019). As a secondary outcome, we used NDI QoL and FD-QoL to evaluate QoL. Although there are studies with contradictory findings on the relationship between FD and QoL, QoL measurement is an important outcome for disease without biological or clinical markers (Talley et al., 1999; El-Serag and Talley, 2003). In this study, after 4 weeks of treatment, the NDI QoL and FD-QoL scores improved in both the groups, but the difference was not significant. To observe improvement in overall FD symptoms, we used VAS, and the VAS scale showed improvement in both groups, but the difference was not significant.

Interestingly, follow-up investigation showed a significantly beneficial effect of BST on FD symptoms, when compared to placebo. Significant improvement observed in VAS score. NDI also showed a pattern of improvement, with a considerable trend toward significance. This indicated that the effect of BST lasted even after the completion of the medication regimen. Collectively, our data suggested that BST did not have any significant effects on FD compared to placebo after 4 weeks of treatment. However, BST showed a statistically significant improvement in the follow-up visit. Considering the prolonged effect of BST in comparison with placebo, this result suggests that BST may not be a palliative treatment but a radical cure on FD. In the safety considerations for both groups, all adverse events were mild and temporary. Moreover, all adverse events, except for urticaria, were related to the prior symptoms of the subjects. The adverse events were, therefore, evaluated as “definitely not related” or “probably not related,” and BST syrup or placebo administration was not stopped for any patient.

Taken together, our results suggest that BST may be moderately helpful in FD via improvement of the overall symptoms, even though FD is a heterogeneous disorder and associated with complicated pathophysiological features, (Talley and Ford, 2015; Enck et al., 2017). These improvements may be due to the diverse functions of BST, such as anti-inflammatory function, enhancing gastric motility and protective function of mucosa (Nakazono et al., 2010; Shaofang et al., 2018; Bai et al., 2019; Jeon et al., 2019; Ji et al., 2021; Zenitani et al., 2021).

In TCM and Korea medicine, it is considered best practice to customize herbal formula for each patient. Even though patients are diagnosed with the same disease, they may be prescribed different or modified herbal medicine according to individual differences and disease states. Therefore, the administration of drugs to patients without considerations of individual differences may not be suitable for herbal medicines and thus, do not show significant statistical differences in RCTs (Yuan and Lin, 2000; Flower et al., 2014). In this trial, we performed RCT based on pattern identification by considering disease states and individual differences, and we identified the role of BST in FD. However, this study included a relatively small number of participants. Thus, further studies with diverse populations are needed to evaluate the efficacy of BST. Additionally, the improvement in FD was not assessed via biological evaluation. Although there are no biological markers of FD, previous studies showed that BST improved GI motility via an increase in the levels of gut regulatory hormones such as somatostatin, motilin, and gastrin (Naito et al., 2002). Therefore, assessing the level of gut regulatory hormones may be an objective indicator for evaluating the efficacy of BST. Moreover, FD is known to be susceptible to placebo effects, and a previous study reported that the placebo response in FD was approximately 30–40% (Talley et al., 2006a; Musial et al., 2007). Therefore, other objective indicators, such as novel nutrient drink test (Lim et al., 2014), gastric emptying scintigraphy (Hafeez et al., 2018), would be preferable for discriminating between the effects of BST and placebo on FD, in the future studies.

## Data Availability

The raw data supporting the conclusion of this article will be made available by the authors, without undue reservation.
